# The effect of inadvertent systemic hypothermia after mechanical thrombectomy in patients with large-vessel occlusion stroke

**DOI:** 10.3389/fneur.2024.1381872

**Published:** 2024-06-04

**Authors:** Kristina auf dem Brinke, Fabian Kück, Ala Jamous, Marielle Ernst, Nils Kunze-Szikszay, Marios-Nikos Psychogios, Ilko L. Maier

**Affiliations:** ^1^Department of Neurology, University Medical Center Göttingen, Göttingen, Germany; ^2^Department of Medical Statistics, University Medical Center Göttingen, Göttingen, Germany; ^3^Department of Neuroradiology, University Medical Center Göttingen, Göttingen, Germany; ^4^Department of Anesthesiology, University Medical Center Göttingen, Göttingen, Germany; ^5^Department of Neuroradiology, Universitätsspital Basel, Basel, Switzerland

**Keywords:** large-vessel occlusion stroke, mechanical thrombectomy, hypothermia, angio suite, functional outcome

## Abstract

**Background and aims:**

Postinterventional hypothermia is a frequent complication in patients with large-vessel occlusion strokes (LVOS) after mechanical thrombectomy (MT). This inadvertent hypothermia might potentially have neuroprotective but also adverse effects on patients’ outcomes. The aim of the study was to determine the rate of hypothermia in patients with LVOS receiving MT and its influence on functional outcome.

**Methods:**

We performed a monocentric, retrospective study using a prospectively derived databank, including all LVOS patients receiving MT between 2015 and 2021. Predictive values of postinterventional body temperature and body temperature categories (hyperthermia (≥38°C), normothermia (35°C–37.9°C), and hypothermia (<35°C)) on functional outcome were analyzed using multivariable Bayesian logistic regression models. Favorable outcome was defined as modified Rankin Scale (mRS) ≤3.

**Results:**

Of the 480 included LVOS patients with MT (46.0% men; mean ± SD age 73 ± 12.9 years), 5 (1.0%) were hyperthermic, 382 (79.6%) normothermic, and 93 (19.4%) hypothermic. Postinterventional hypothermia was significantly associated with unfavorable functional outcome (mRS > 3) after 90 days (OR 2.06, 95% CI 1.01–4.18, *p* = 0.045). For short-term functional outcome, patients with hypothermia had a higher discharge NIHSS (OR 1.38, 95% CI 1.06 to 1.79, *p* = 0.015) and a higher change of NIHSS from admission to discharge (OR 1.35, 95% CI 1.03 to 1.76, *p* = 0.029).

**Conclusion:**

Approximately a fifth of LVOS patients in this cohort were hypothermic after MT. Hypothermia was an independent predictor of unfavorable functional outcomes. Our findings warrant a prospective trial investigating active warming during MT.

## 1 Introduction

Stroke is one of the leading causes for death and disability worldwide. The introduction of mechanical thrombectomy (MT) for patients with large-vessel occlusion stroke (LVOS) significantly improved functional outcome and is currently considered the gold standard in this subgroup of stroke patients (1). However, still less than 50% of LVOS patients treated with MT, even despite fast and complete recanalization, achieve functional independence at 90 days and the 90-day mortality rate is approximately 15% (1). Multiple peri-interventional factors influence functional outcome in LVOS patients, leading to a continued development to improve pre-and intrahospital management and to investigate neuroprotective measures in this important subgroup of stroke patients.

Therapeutic hypothermia (TH) has been shown to improve neurological recovery in patients with generalized brain ischemia after out-of-hospital cardiac arrest (2) and is recommended by guidelines in this patient group (3). The effects of TH are multimechanistic, affecting almost every molecular and cellular pathway known to lead to cell death (4). However, the role of TH in the focal brain ischemia associated with stroke remains unclear. Clinical trials examining systemic cooling techniques of stroke patients have failed to show any benefit (5, 6). A meta-analysis including 12 studies did not observe a greater overall improvement in functional outcome in patients with TH in acute ischemic stroke than standard of care, although some studies demonstrated a shift toward better outcomes (7). This might be due to the higher incidence of complications observed in the TH groups, mainly in form of infections and cardiac complications (e.g., bradyarrhythmia) (7). Therefore, neuroprotective effect might be outweighed by cardiac dysfunction and hemodynamic imbalance (8) by increasing catecholamine release and myocardial oxygen demand (9). In addition, the efficacy of TH has been shown to depend on the duration, timing, depth, and kind of application of hypothermia, all of which are not clear in focal cerebral ischemia (10).

Postinterventional hypothermia is a frequent complication in stroke patients after MT, which always is performed in an emergency setting (11). General anesthesia, prolonged MT times, and the fact that the patient lies motionless—in many cases without transurethral catheters—in the angio suite can lead to a quick drop in the patient’s core temperature. In this respect, as many mechanical thrombectomies are performed quickly (12), active warming systems are not widely used. This inadvertent hypothermia might potentially have neuroprotective but also detrimental effects on patients’ outcomes (e.g., increased infection rates or hypothermia-associated coagulopathy). Recent studies analyzing the effect of body temperature on functional outcomes of MT patients were inconclusive. While a study by Hartmann et al. suggested that inadvertent hypothermia after MT had no influence on functional outcome or mortality but is associated with an increased rate of pneumonia and bradyarrhythmia (13), a study by Xu et al. found that lower intraoperative body temperature during MT was independently associated with improved neurological outcome (14).

Given this inconclusive data, this study aimed to determine the rate of hypothermia in LVOS patients receiving MT and to investigate its effect on functional outcomes in a large, prospectively derived stroke database.

## 2 Materials and methods

### 2.1 Patient population

We performed a monocentric, retrospective study using a prospectively derived databank including all LVOS patients receiving MT at University Medical Center Göttingen, Germany. Available data of patients enrolled between 2015 and 2022 were analyzed. Inclusion criteria were diagnosis of anterior circulation LVOS (proximal internal carotid artery (ICA), ICA bifurcation, and medial cerebral artery (MCA) M1, M2, and M3) treated with mechanical thrombectomy and an age ≥ 18 years. Patients with an incomplete set of data included in the registry were excluded. The frontline thrombectomy strategy and access were at the discretion of the treating neurointerventionalist. All data were assessed prospectively during the in-hospital stay of every patient by local senior neuroradiologists and neurologists, including time metrics, peri-procedural management data, and outcome assessment at discharge and after 90 days. Stroke severity was assessed by the National Institutes of Health Stroke Scale (NIHSS). The degree of disability was rated by the modified Rankin Scale (mRS) (15). Early signs of infarction on imaging were assessed using the Alberta Stroke Program Early CT Score (ASPECT) (16). Initial imaging was performed using native computer tomography (CT), CT angiography, and perfusion. Reperfusion success was measured using the modified thrombolysis in cerebral infarction (mTICI) scale (17). mTICI 2b-3 was regarded as a successful reperfusion. All patients were admitted to a neurological intensive care unit (ICU) immediately after MT, on which vital signs were recorded automatically by a patient data management system (IntelliSpace Critical Care and Anesthesia (ICCA), Philips, Germany). Core body temperature recordings were checked for consistency in every patient included in this study.

### 2.2 Assessment of temperature readings after endovascular therapy

Temperature measurements upon arrival at the neuro-ICU and within the first 12 h after arrival were analyzed. Body temperature was measured continuously by a urinary catheter with a temperature probe. All temperature measurements were reported in the electronic health record. We defined normothermia as temperature values between 35°C and 37.9°C, hyperthermia as temperature values greater than or equal to 38°C, and hypothermia as temperature values less than 35°C.

### 2.3 Study endpoints

The primary endpoint of this study was a favorable functional outcome at 90 days after stroke, defined as an mRS score ≤ 3. Secondary outcomes were mRS and NIHSS at discharge as well as relative change of NIHSS from admission to discharge (= difference of NIHSS at discharge and NIHSS at admission divided by NIHSS at admission).

### 2.4 Statistical analysis

All variables have been summarized using absolute and relative frequencies, mean ± standard deviation or median (IQR), as appropriate, for the whole cohort and different groups. For two patients with NIHSS = 0 at admission, the relative change could not be computed and was thus considered missing.

For the comparison of patients with mRS ≤ 3 and mRS > 3 at discharge and 90 days after discharge, we applied Fisher’s exact test for nominal variables, Welch’s *t*-test for age, time metrics as well as count data, and the Brunner–Munzel test for all other numeric and ordinal variables. For the comparison of patients with and without hypothermia, we used the Brunner–Munzel test.

We performed a receiver operating characteristic (ROC) analysis for mRS > 3 and reported the area under the ROC curve with the corresponding confidence interval, which was computed using the DeLong method.

We fitted multivariable Bayesian logistic regression models on the complete cases to analyze the influence of hypothermia on the probability of mRS > 3 at discharge as well as 90 days after discharge using normal priors with a scale of 2.5. For modeling the NIHSS, we first log(*x* + 1)-transformed the NIHSS values to satisfy model assumptions. We applied multivariable linear regression models on the complete cases to evaluate the effect of hypothermia on the NIHSS at discharge and on the difference of (log(*x* + 1)-transformed) NIHSS between admission and discharge.

The significance level was set to *α* = 5% for all statistical tests. Due to the exploratory nature of this study, no adjustment for multiple testing was applied. All analyses were performed with the statistical programming environment R (version 3.6.2; R Core Team 2019) using the R-packages nparcomp (version 3.0) for the Brunner–Munzel test, pROC (version 1.18.0) for the ROC analysis, arm (version 1.13.1) for Bayesian logistic regression models, and ggeffects (version 1.1.4) for the computation and visualization of marginal effect.

## 3 Results

### 3.1 Baseline characteristics

At the time of data analysis, the databank included 1,297 cases. After discarding cases with missing data and patients with posterior circulation ischemic stroke, 480 patients remained for the analysis of the primary endpoint.

Among the 480 study patients, 221 patients (46.0%) were men, the mean age was 73 ± 12.9 years, and the median NIHSS score at admission was 15 (IQR 11 to 19). MT was performed with general anesthesia in 292 patients (75.3%) and with conscious sedation in 96 patients (24.7%); 360 patients (85.9%) achieved successful recanalization (modified Thrombolysis in Cerebral Infarction 2b-3), and 20 (4.4%) had symptomatic intracerebral hemorrhage. At 3 months, 223 (46.5%) patients had a favorable functional outcome (mRS ≤ 3). The baseline characteristics of patients with and without favorable functional outcomes are shown in Table 1.

**Table 1:** Baseline characteristics of LVOS patients receiving MT with mRS ≤ 3 and mRS > 3 after 90 days.

	All patients (*n* = 480)	mRS ≤ 3 (*n* = 223)	mRS > 3 (*n* = 257)	*p*-value
Demographics and clinical data				
Male sex (*n*, %)	221 (46.0%)	108 (48.4%)	113 (44.0%)	0.234
Age (mean ± SD)	73.0 ± 12.9	68.5 ± 13.3	76.8 ± 11.1	<0.001
Baseline NIHSS (median score, IQR)	15 (11; 19)	14 (8; 18)	16 (12; 20)	<0.001
Medical history				
Diabetes mellitus (*n*, %)	143 (30.2%)	54 (24.3%)	89 (35.5%)	0.009
Arterial hypertension (*n*, %)	381 (79.9%)	159 (71.6%)	222 (87.1%)	<0.001
History of AF (*n*, %)	219 (46.5%)	88 (40.4%)	131 (51.8%)	0.016
Dyslipoproteinemia (*n*, %)	231 (48.9%)	118 (53.4%)	113 (45.0%)	0.080
Previous and current smoking (*n*, %)	86 (18.7%)	52 (24.1%)	34 (13.9%)	0.006
Prior stroke (*n*, %)	24 (15.4%)	8 (10.7%)	16 (19.8%)	0.127
Anesthesia				0.006
Conscious sedation (*n*, %)	96 (24.7%)	53 (31.9%)	43 (19.4%)	
General anesthesia (*n*, %)	292 (75.3%)	113 (68.1%)	179 (80.6%)	
Time metrics				
Onset-to-needle time (mean min ± SD)	212 ± 224	217 ± 299	207 ± 110	0.662
Onset/last seen well-to-groin time (mean h ± SD)	5.14 ± 5.69	4.54 ± 5.58	5.67 ± 5.74	0.034
Onset-to-first TICI≥2b (mean min ± SD)	257 ± 233	260 ± 306	254 ± 107	0.804
Groin-to-first TICI≥2b (mean min ± SD)	47.5 ± 29.9	43.5 ± 30.2	51.5 ± 29.0	0.006
Imaging data				
ASPECTS at baseline (median points, IQR)	8 (7; 9)	9 (8; 9)	8 (6; 9)	<0.001
Site of vessel occlusion				0.070
Proximal ICA (*n*, %)	32 (6.7%)	15 (6.7%)	17 (6.6%)	
ICA bifurcation (*n*, %)	122 (25.4%)	46 (20.6%)	76 (29.6%)	
MCA, M1 segment (*n*, %)	246 (51.2%)	116 (52.0%)	130 (50.6%)	
MCA, M2 segment (*n*, %)	75 (15.6%)	44 (19.7%)	31 (12.1%)	
MCA, M3 segment (*n*, %)	5 (1.0%)	2 (0.9%)	3 (1.2%)	
Procedural and imaging outcomes				
Number of aspirations (mean ± sd)	1.99 ± 1.59	1.91 ± 1.64	2.05 ± 1.54	0.368
Number of passes (mean ± sd)	1.71 ± 1.07	1.53 ± 0.77	1.85 ± 1.24	0.003
Final TICI score (median, IQR)	2c (2b67; 3)	2c (2b67; 3)	2c (2b50; 3)	< 0.001
Successful recanalization	360 (85.9%)	178 (95.2%)	182 (78.4%)	< 0.001
Hemorrhage present	79 (16.7%)	20 (9.1%)	59 (23.4%)	< 0.001
sICH	20 (4.4%)	2 (0.9%)	18 (7.4%)	< 0.001
Functional outcomes				
NIHSS discharge (median points, IQR)	5 (2; 12)	2 (1; 5)	13 (7; 18)	< 0.001
relative change of NIHSS (median, IQR)	-0.67 (−0.86; 0.20)	−0.81 (−0.93; 0.67)	−0.20 (−0.50; 0.00)	< 0.001
mRS at discharge (median score, IQR)	4 (0; 6)	1 (0; 5)	5 (0; 6)	< 0.001
mRS after 90 days (median score, IQR)	4 (0; 6)	1 (0; 3)	6 (4; 6)	
Mortality at 90 days (*n*, %)	140 (29.2%)	0 (0.0%)	140 (54.5%)	

LVOS, large-vessel occlusion stroke; MT, mechanical thrombectomy; mRS, modified Rankin Scale; SD, standard deviation; NIHSS, National Institute of Health Stroke Scale; IQR, interquartile range; AF, atrial fibrillation; ASPECTS, Alberta stroke program early CT score; ICA, internal carotid artery; MCA, medial cerebral artery; TICI, thrombolysis in cerebral infarction; successful recanalization, TICI ≥ 2b on final angiogram; sICH, symptomatic intracerebral hemorrhage.

Patients with favorable functional outcomes after 90 days had significantly lower NIHSS at admission (*p* < 0.001) and were less likely to have cardiovascular comorbidities such as diabetes mellitus (24.3% vs. 35.5%, *p* = 0.009), arterial hypertension (71.6% vs. 87.1%, *p* < 0.001), and atrial fibrillation (40.4% vs. 51.8%, *p* = 0.016) as well as were significantly younger (68.5 ± 13.3 vs. 76.8 ± 11.1, *p* < 0.001). There was no significant difference in sex (48.4% vs. 44.0% male, *p* = 0.359) and location of vessel occlusion (*p* = 0.070).

### 3.2 Postinterventional body temperature

On admission to the neuro-ICU, 382 patients (79.6%) were normothermic, 93 patients (19.4%) were hypothermic, and 5 patients (1.0%) were hyperthermic. The median increase in body temperature from admission to 12-h length of stay was 1.2°C (IQR 0.4 to 2.0) (Supplementary Table S1).

### 3.3 Unadjusted analysis

**Figure 1:**
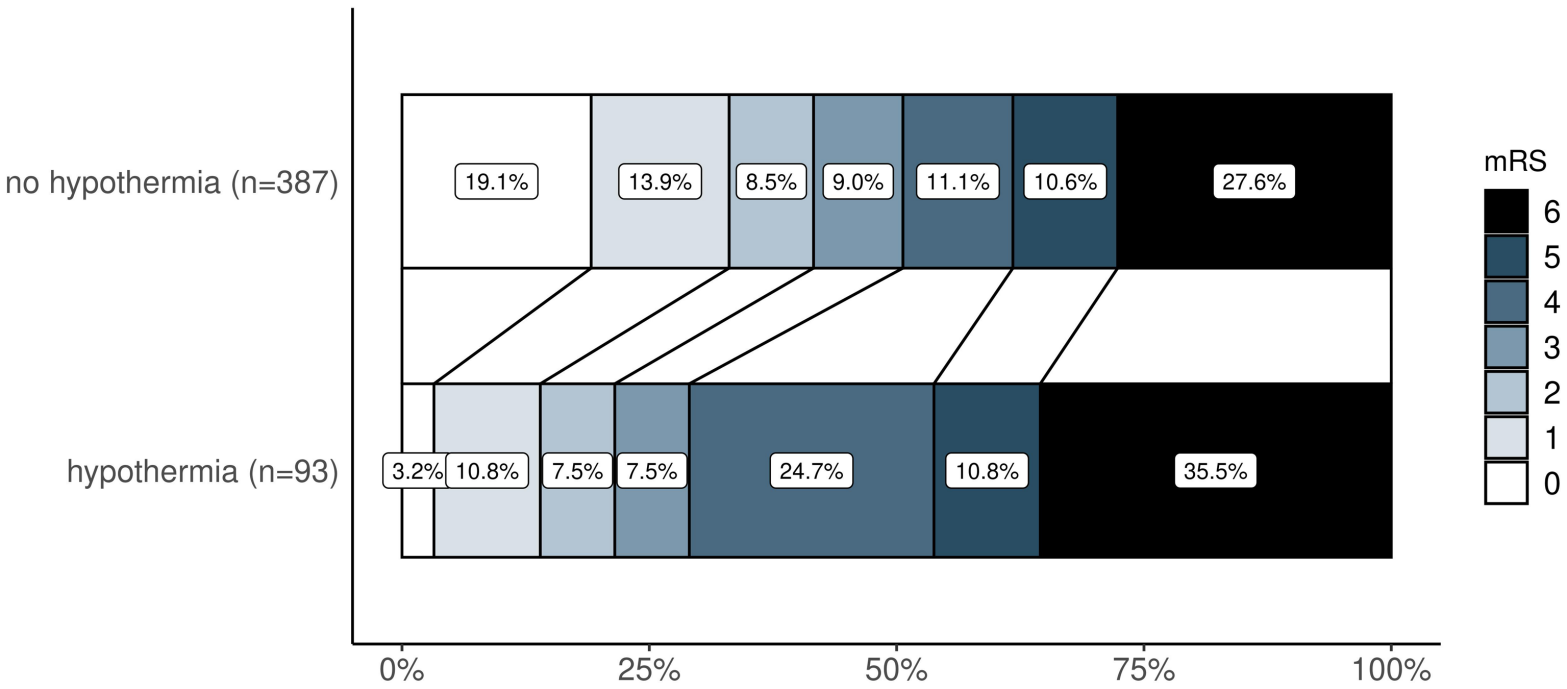
Ordinal shift analysis—comparison of mRS distribution after 90 days between patients with and without hypothermia. Legend (right) demonstrates how mRS is represented by colors from 0 (in white) to 6 (in black). mRS, modified Rankin Scale.

In patients with favorable functional outcomes after 90 days, median temperature on admission to neuro-ICU was higher (36.0 [IQR 35.4 to 36.4] vs. 35.7 [IQR 34.9 to 36.4], *p* = 0.006), and hypothermia (12.1% vs. 25.7%, *p* < 0.001) as well as hyperthermia (0.4% vs. 1.6%, *p* = 0.379) were less frequent. The change of temperature from admission to temperature after 12 h was significantly higher in patients with unfavorable outcomes (median 1.0 [IQR 0.3 to 1.8] vs. 1.3 [IQR 0.6 to 2.2], *p* = 0.003). Temperature data of patients with favorable and unfavorable outcomes after 90 days are shown in Supplementary Table S1. There was a shift toward better functional outcomes on the mRS after 90 days favoring patients without hypothermia (Figure 1). In ROC analysis, the optimal cutoff temperature after MT according to the Youden index for an unfavorable outcome at 90 days was 35.0°C and for an unfavorable outcome at discharge 35.8°C (Supplementary Figure S2).

In patients with hypothermia after MT, NIHSS at discharge was significantly higher (median 9 [IQR 4 to 16] vs. 5 [IQR 2 to 12], *p* < 0.001) and relative improvement in NIHSS was smaller (median relative change-0.40 [IQR −0.76 to −0.11] vs. -0.67 [IQR −0.86 to −0.17], *p* = 0.005) (Supplementary Table S2).

### 3.4 Adjusted analysis

Multivariable regression models demonstrated a significantly higher chance for an unfavorable functional outcome after 90 days in patients with postinterventional hypothermia (OR 2.06, 95% CI 1.01 to 4.18, *p* = 0.045, Table 2). Other independent predictors of an unfavorable outcome at 90 days were higher age (OR 1.03, 95% CI 1.00 to 1.06, *p* = 0.025), arterial hypertension (OR 2.72, 95% CI 1.23 to 6.00, *p* = 0.013), and low aspect score (OR 0.78, 95% CI, 0.64 to 0.97, *p* = 0.024) (Table 2).

**Table 2:** Multivariable logistic regression model for an unfavorable functional outcome (mRS > 3) after 90 days including postinterventional hypothermia.

	*N*	No. of events	OR	95% CI	*p*-value
Postinterventional hypothermia	58	39	2.06	(1.01, 4.18)	0.045
Age	231	122	1.03	(1.00, 1.06)	0.025
Baseline NIHSS	231	122	1.05	(1.00, 1.10)	0.072
Diabetes mellitus	68	41	1.62	(0.82, 3.20)	0.161
Arterial hypertension	177	104	2.72	(1.23, 6.00)	0.013
History of AF	100	58	1.06	(0.56, 1.99)	0.867
Dyslipoproteinemia	110	53	0.63	(0.34, 1.19)	0.155
General anesthesia	172	97	1.49	(0.75, 2.99)	0.254
Onset/last seen well-to-first TICI≥2b	231	122	1.07	(1.00, 1.15)	0.055
ASPECTS at baseline	231	122	0.78	(0.64, 0.97)	0.024
Successful recanalization	220	113	0.20	(0.04, 1.10)	0.063
sICH	4	4	7.44	(0.37, 152)	0.189

mRS, modified Rankin Scale; OR, odds ratio; CI, confidence interval; NIHSS, National Institute of Health Stroke Scale; AF, atrial fibrillation; TICI, thrombolysis in cerebral infarction; ASPECTS, Alberta stroke program early CT score; successful recanalization, TICI ≥ 2b on final angiogram; sICH, symptomatic intracerebral hemorrhage.

For short-term functional outcomes, there was a trend toward a higher chance for an unfavorable discharge mRS in patients with hypothermia (OR 1.78, 95% CI 0.91 to 3.47, *p* = 0.088) and significant results for discharge NIHSS (OR 1.38, 95% CI 1.06 to 1.79, *p* = 0.015) as well as relative change of NIHSS from admission to discharge (OR 1.35, 95% CI 1.03 to 1.76, *p* = 0.029) (Supplementary Tables S3–S5, Supplementary Figure S1).

The difference from postinterventional body temperature to temperature after 12 h was a significant predictor of poorer functional outcome after 90 days (OR 1.28, 95% CI 1.02 to 1.60, *p* = 0.029) (Table 3) and at discharge (OR 1.31, 95% CI 1.05 to 1.63, *p* = 0.016) (Supplementary Table S6).

**Table 3:** Multivariable logistic regression model for an unfavorable functional outcome (mRS > 3) after 90 days including postinterventional change of body temperature.

	*N*	No. of events	OR	95% CI	*p*-value
∆ Temperature	231	122	1.28	(1.02, 1.60)	0.029
Postinterventional body temperature	231	122	0.97	(0.73, 1.29)	0.834
Age	231	122	1.03	(1.00, 1.06)	0.022
Baseline NIHSS	231	122	1.04	(0.99, 1.10)	0.119
Diabetes mellitus	68	41	1.47	(0.74, 2.91)	0.271
Arterial hypertension	117	104	2.60	(1.17, 5.76)	0.018
History of AF	100	58	1.03	(0.54, 1.95)	0.926
Dyslipoproteinemia	110	53	0.63	(0.33, 1.20)	0.155
General anesthesia	172	97	1.45	(0.72, 2.94)	0.297
Onset/last seen well-to-first TICI≥2b	231	122	1.07	(1.00, 1.15)	0.056
ASPECTS at baseline	231	122	0.73	(0.59, 0.91)	0.006
Successful recanalization	220	113	0.22	(0.04, 1.20)	0.079
sICH	4	4	7.72	(0.39, 154)	0.179

mRS, modified Rankin Scale; OR, odds ratio; CI, confidence interval; ∆ Temperature, temperature 12 h after admission to intensive care unit minus postinterventional body temperature; NIHSS, National Institute of Health Stroke Scale; AF, atrial fibrillation; TICI, thrombolysis in cerebral infarction; ASPECTS, Alberta stroke program early CT score; successful recanalization, TICI ≥ 2b on final angiogram; sICH, symptomatic intracerebral hemorrhage.

## 4 Discussion

Postinterventional hypothermia is a frequent complication in LVOS patients after MT. This inadvertent hypothermia could potentially have neuroprotective but also detrimental effects on patients’ functional outcomes.

In this study, hypothermia after MT was associated with an unfavorable functional outcome. We found a significant association between postinterventional hypothermia and an unfavorable functional outcome at 90 days. In addition, there was a trend for unfavorable functional outcome at discharge and significant results for the NIHSS score at discharge as well as relative change in NIHSS. Our study, therefore, does not support the hypothesis that inadvertent hypothermia in LVOS patients may have neuroprotective effects but might contribute to worse functional outcomes despite successful recanalization. While moderate therapeutic hypothermia has been shown to improve clinical outcomes in patients with cardiac arrest (2, 18) and in animal stroke models (19), clinical studies have failed to show a beneficial effect of induced hypothermia in human stroke patients (5–7, 20, 21). This might be due to the fact that many patients with LVOS have cardiovascular risk factors, and therapeutic hypothermia may further disrupt hemodynamic balance and deteriorate cardiac function (8). Conversely, patients of advanced age and those with overweight or chronic diseases are more likely to develop perioperative hypothermia (22). That is why we adjusted for cardiovascular risk factors as possible confounders.

Inadvertent hypothermia is a common side effect in patients undergoing surgery. It can occur as a result of the suppression of central mechanisms of temperature regulation due to anesthesia and prolonged exposure of large skin surfaces to cold temperatures in operating rooms (23). Perioperative hypothermia has negative effects on coagulation, blood loss and transfusion requirements, metabolization of drugs, surgical site infections, and discharge from the post-anesthesia care unit (24). Active body surface warming (ABSW) systems are effective in maintaining physiological normothermia and are used to prevent adverse clinical outcomes (25). The results of our study underline the importance of ABSW systems to maintain normothermia even with short thrombectomy times.

In the randomized SIESTA trial, hypothermia was associated with the performance of MT under general anesthesia (11). In our study, general anesthesia was associated with an unfavorable functional outcome at 90 days. At the moment, the impact of the type of anesthetic technique on neurological outcomes is still under debate (26). As general anesthesia was associated with worse functional outcomes of MT patients in some other cohorts as well (27), a potential beneficial effect of hypothermia may have been confounded by the performance of general anesthesia in our study. Therefore, we corrected for general anesthesia as a confounder.

In a recent observational study by Hartmann et al. (13) including 416 patients with anterior circulation large-vessel occlusion treated with EVT, approximately half of the patients (50.2%) were hypothermic (<36.0°C; median body temperature 35.2°C) and half were normothermic (36.0°C to 37.5°C; median body temperature 36.4°C) after MT. Patients with temperature > 37.5°C after EVT were excluded. Hypothermia after EVT was associated with higher age and with general anesthesia for EVT, while there was no association with the duration of the procedure. The multivariate outcome analysis could not demonstrate an association of hypothermia with favorable functional outcome (mRS score < 3 at 3 months), which aligns with our results. More hypothermic patients suffered from pneumonia (36.4% vs. 25.6%, *p* = 0.02) and bradyarrhythmia (52.6% vs. 16.4%, *p* < 0.001), whereas there was no significant difference in thromboembolic events.

As in our study, Hartmann et al. address inadvertent hypothermia and not induced hypothermia as a possible neuroprotective approach. In both studies, inadvertent hypothermia might be a surrogate marker for worse medical pre-condition and more severely affected stroke patients, despite correcting for possible confounders in multivariate analysis. This may have restricted the ability to detect a possible neuroprotective effect.

For acute ischemic stroke patients with LVOS, EVT is a highly effective therapy (1). Therefore, additional therapy effects in EVT patients might be difficult to find out. To avoid the side effects of systemic hypothermia, selective brain cooling using endovascular cooling catheters combined with endovascular reperfusion might be a promising approach (28). For selective intra-arterial cold saline infusion, feasibility and safety were demonstrated in a pilot trial by Chen et al. (29).

The negative influence of higher body temperature on post-procedural infarction growth and functional outcome of MT patients has already been described (30, 31). Consistently, in our study rates of postinterventional hyperthermia were higher in patients with unfavorable functional outcomes. However, infarct volume and expansion were not available in our dataset.

An important limitation of the study is the demonstration of an association; it is not possible to show causality. Hypothermia might be an epiphenomenon of worse functional outcomes caused by an imbalance in comorbidities and/or nutritional status (cachexia, sarcopenia, pre-stroke functional status). In addition, hypothermia might be an indicator of longer procedural delays and durations of mechanical thrombectomy. Therefore, we corrected for cardiovascular risk factors and treatment times. Another limitation is the lack of a control group with active warming of the patients and the monocentric design of the study. The strength of the study is the detailed analysis of a prospectively derived databank and the control for multiple possible confounders.

Further studies are needed to evaluate the effects of the combination of targeted temperature management and MT in acute ischemic stroke patients in randomized clinical trials. Until then, since hypothermia, as well as hyperthermia, were shown to be associated with an unfavorable clinical outcome, we suggest maintaining strict normothermia in LVOS patients treated with MT using active body surface warming (ABSW) systems.

## Data availability statement

The raw data supporting the conclusions of this article will be made available by the authors, without undue reservation.

## Ethics statement

The studies involving humans were approved by Ethics Committee of University Medicine Göttingen (reference number: 13/7/15). The studies were conducted in accordance with the local legislation and institutional requirements. The participants provided their written informed consent to participate in this study.

## Author contributions

Ka: Writing – review & editing, Writing – original draft, Visualization, Project administration, Methodology, Investigation, Formal analysis, Data curation. FK: Writing – review & editing, Visualization, Methodology, Investigation, Data curation. AJ: Writing – review & editing, Data curation. ME: Writing – review & editing, Data curation. NK-S: Writing – review & editing. M-NP: Writing – review & editing. IM: Writing – review & editing, Supervision, Project administration, Methodology, Investigation, Funding acquisition, Formal analysis, Data curation, Conceptualization.
