# Supplementary material for: The effect of inadvertent systemic hypothermia after mechanical thrombectomy in patients with large-vessel occlusion stroke
Source: Front Neurol. 2024 Jun 4;15:1381872. doi: 10.3389/fneur.2024.1381872 (PMC11188377; doi:10.3389/fneur.2024.1381872)
Supplement: Supplementary file 1 [file Data_Sheet_1.PDF]

Supplementary Material

1 Supplementary Tables

Supplemental Table 1: Temperature Data of LVOS patients receiving MT with mRS≤3 and mRS>3 after 90 days.

	All patients (n=637)	mRS≤3 (n=308)	mRS>3 (n=329)	p-value
**Postinterventional body temperature categories**				<0.001
Hypothermia	93 (19.4%)	27 (12.1%)	66 (25.7%)	
Normothermia	382 (79.6%)	195 (87.4%)	187 (72.8%)	
Hyperthermia	5 (1.0%)	1 (0.4%)	4 (1.6%)	
**Temperature data**				
Postinterventional body temperature (°C) (median, IQR)	35.9 (35.2; 36.4)	36.0 (35.4; 36.4)	35.7 (34.9; 36.4)	0.006
Temperature after 12 h (°C) (median, IQR)	37.0 (36.6; 37.5)	37.0 (36.6; 37.5)	37.0 (36.6; 37.5)	0.571
Δ Temperature (°C) (median, IQR)	1.2 (0.4; 2.0)	1.0 (0.3; 1.8)	1.3 (0.6; 2.2)	0.003

LVOS, large vessel occlusion stroke; MT, mechanical thrombectomy; mRS, modified Rankin Scale; Δ Temperature, Temperature after 12 h minus postinterventional body temperature.

Supplemental Table 2: Comparison of NIHSS of patients with and without hypothermia.

	All patients (n=555)	No hypothermia (n=266)	Hypothermia (n=289)	p-value
NIHSS at discharge (median, IQR)	5 (2.0; 13.0)	5 (2.0; 12.0)	9 (3.5; 15.5)	<0.001
Relative change of NIHSS (median, IQR)	-0.62 (-0.85; -0.14)	-0.67 (-0.86; -0.17)	-0.40 (-0.76; -0.11)	0.005

NIHSS, National Institute of Health Stroke Scale; Relative change of NIHSS, difference of NIHSS at discharge and NIHSS at admission divided by NIHSS at admission.

Supplemental Table 3: Multivariable logistic regression model for an unfavorable functional outcome (mRS>3) at discharge including postinterventional hypothermia.

	N	No. of events	OR	95% CI	p-value
Postinterventional hypothermia	61	36	1.78	(0.91, 3.47)	0.088
Age	245	113	1.02	(0.99, 1.04)	0.194
Baseline NIHSS	245	113	1.06	(1.01, 1.11)	0.022
Diabetes mellitus	72	37	1.56	(0.81, 2.99)	0.180
Arterial hypertension	190	93	1.84	(0.85, 4.00)	0.120
History of AF	106	51	0.98	(0.52, 1.81)	0.936
Dyslipoproteinemia	120	48	0.66	(0.36, 1.21)	0.175
General anesthesia	179	91	1.72	(0.88, 3.39)	0.114
Onset/Last seen well-to-first TICI≥2b	245	113	1.04	(0.98, 1.11)	0.219
ASPECTS at baseline	245	113	0.68	(0.56, 0.84)	< 0.001
Successful recanalization	234	104	0.16	(0.03, 0.83)	0.029
sICH	4	4	8.54	(0.40, 180)	0.166

mRS, modified Rankin Scale; OR, Odds Ratio; CI, Confidence Interval; NIHSS, National Institute of Health Stroke Scale; AF, atrial fibrillation; TICI, thrombolysis in cerebral infarction; ASPECTS, Alberta stroke program early CT score; successful recanalization, TICI≥2b on final angiogram; sICH, symptomatic intracerebral hemorrhage.

Supplemental Table 4: Multivariable linear regression model for NIHSS at discharge. Note that NIHSS was log(x+1)-transformed so that estimated percentage difference is given by $100 \cdot (\exp(\beta)-1)$.

	N	exp(β)	95% CI	p-value
Postinterventional hypothermia	52	1.38	(1.06, 1.79)	0.015
Age	214	1.00	(0.99, 1.01)	0.992
Baseline NIHSS	214	1.05	(1.03, 1.07)	< 0.001
Diabetes mellitus	62	1.24	(0.96, 1.59)	0.098
Arterial hypertension	164	1.31	(0.98, 1.74)	0.068
History of AF	94	1.07	(0.84, 1.36)	0.580
Dyslipoproteinemia	109	0.89	(0.71, 1.12)	0.321
General anesthesia	154	1.12	(0.87, 1.43)	0.370
Onset/Last seen well-to-first TICI≥2b	214	1.03	(1.01, 1.06)	0.011
ASPECTS at baseline	214	0.88	(0.81, 0.95)	0.001
Successful recanalization	208	0.41	(0.21, 0.81)	0.010
sICH	1	1.63	(0.31, 8.51)	0.561

NIHSS, National Institute of Health Stroke Scale; CI, Confidence Interval; AF, atrial fibrillation; TICI, thrombolysis in cerebral infarction; ASPECTS, Alberta stroke program early CT score; successful recanalization, TICI≥2b on final angiogram; sICH, symptomatic intracerebral hemorrhage.

Supplemental Table 5: Multivariable linear regression model for change of NIHSS from admission to discharge. Note that NIHSS was log(x+1)-transformed so that estimated percentage differences of relative NIHSS changes from admission to discharge are given by 100·(exp(β)-1).

	N	exp(β)	95% CI	p-value
Postinterventional hypothermia	52	1.35	(1.03, 1.76)	0.029
Age	214	1.00	(0.99, 1.01)	0.892
Baseline NIHSS	214	0.97	(0.96, 0.99)	0.009
Diabetes mellitus	62	1.31	(1.01, 1.69)	0.044
Arterial hypertension	164	1.33	(0.99, 1.78)	0.062
History of AF	94	1.09	(0.85, 1.39)	0.591
Dyslipoproteinemia	109	0.90	(0.71, 1.14)	0.365
General anesthesia	154	1.16	(0.90, 1.50)	0.246
Onset/Last seen well-to-first TICI≥2b	214	1.03	(1.00, 1.05)	0.020
ASPECTS at baseline	214	0.89	(0.82, 0.97)	0.005
Successful recanalization	208	0.38	(0.19, 0.75)	0.006
sICH	1	1.95	(0.35, 10.8)	0.441

NIHSS, National Institute of Health Stroke Scale; CI, Confidence Interval; AF, atrial fibrillation; TICI, thrombolysis in cerebral infarction; ASPECTS, Alberta stroke program early CT score; successful recanalization, TICI≥2b on final angiogram; sICH, symptomatic intracerebral hemorrhage.

Supplemental Table 6: Multivariable logistic regression model for an unfavorable functional outcome (mRS >3) at discharge including postinterventional change of body temperature.

	N	No. of events	OR	95% CI	p-value
Δ Temperature	245	113	1.31	(1.05, 1.63)	0.016
Postinterventional body temperature	245	113	0.83	(0.62, 1.10)	0.191
Age	245	113	1.02	(0.99, 1.04)	0.198
Baseline NIHSS	245	113	1.05	(1.00, 1.11)	0.054
Diabetes mellitus	72	37	1.48	(0.76, 2.88)	0.250
Arterial hypertension	190	93	1.87	(0.84, 4.13)	0.122
History of AF	106	51	0.95	(0.51, 1.79)	0.875
Dyslipoproteinemia	120	48	0.64	(0.34, 1.18)	0.152
General anesthesia	179	91	1.50	(0.75, 3.02)	0.253
Onset/Last seen well-to-first TICI≥2b	245	113	1.04	(0.98, 1.11)	0.212
ASPECTS at baseline	245	113	0.64	(0.51, 0.80)	< 0.001
Successful recanalization	234	104	0.16	(0.03, 0.86)	0.032
sICH	4	4	8.94	(0.43, 184)	0.154

mRS, modified Rankin Scale; OR, Odds Ratio; CI, Confidence Interval; Δ Temperature, Temperature after 12 h minus postinterventional body temperature; NIHSS, National Institute of Health Stroke Scale; AF, atrial fibrillation; TICI, thrombolysis in cerebral infarction; ASPECTS, Alberta stroke program early CT score; successful recanalization, TICI≥2b on final angiogram; sICH, symptomatic intracerebral hemorrhage.

2 Supplementary Figures

Supplemental Figure 1

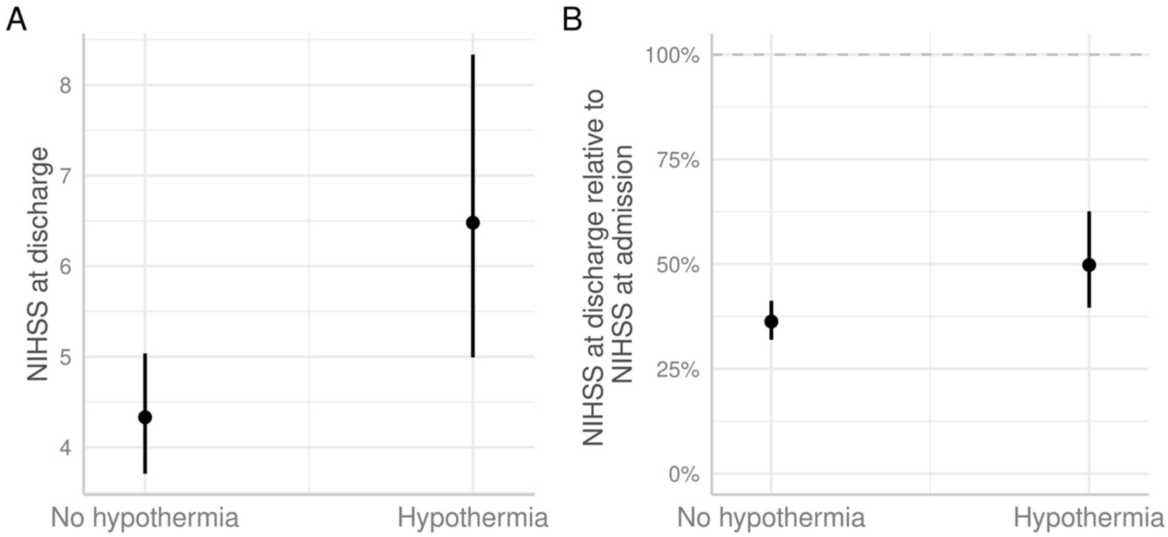

A) Estimated marginal effects of postinterventional hypothermia and corresponding 95% confidence intervals. Note that the estimated marginal effects are expected to be close to the median NIHSS at discharge, which is lower than the mean due to the skewed distribution of NIHSS values at discharge. B) Estimated NIHSS at discharge relative to NIHSS at admission and corresponding 95% confidence intervals. Note that one was added to the NIHSS to avoid division by zero. Note further that 100% corresponds to the NIHSS value at admission. NIHSS, National Institute of Health Stroke Scale.

Supplemental Figure 2

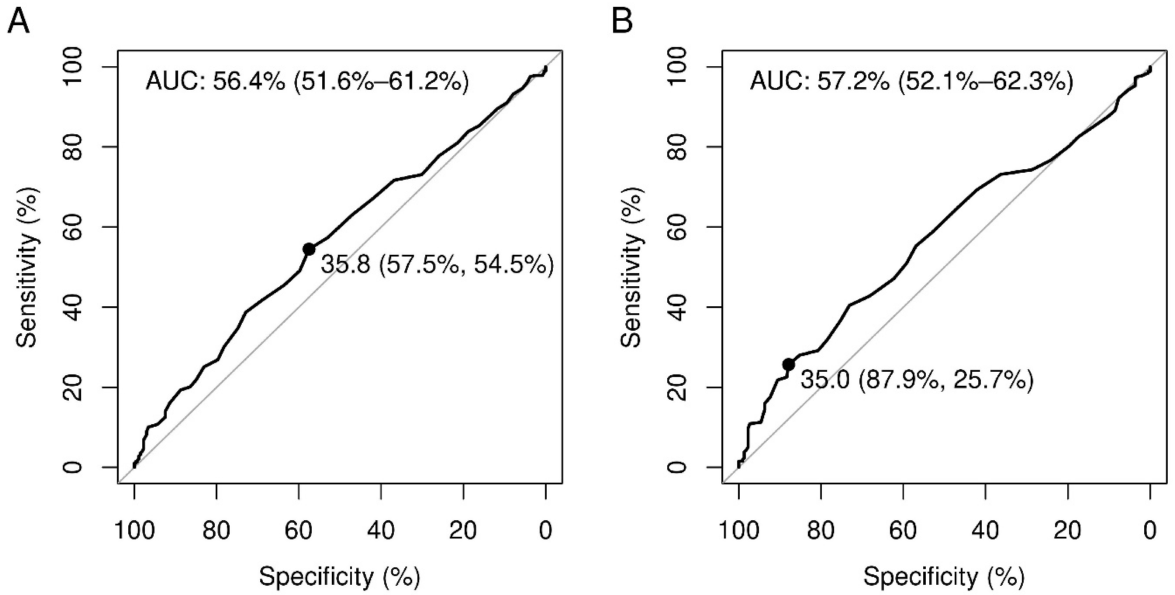

A) ROC curve for postinterventional body temperature and mRS at discharge and the optimal cut-off according to the Youden index (with corresponding sensitivity and specificity values) and the AUC (with the corresponding 95% confidence interval). mRS, modified Rankin Scale. B) ROC curve for postinterventional body temperature and mRS at 90 days, the optimal cut-off according to the Youden index (with corresponding sensitivity and specificity values) and the AUC (with the corresponding 95% confidence interval).
